# Genome-wide discovery of maternal effect variants

**DOI:** 10.1186/1753-6561-3-s7-s19

**Published:** 2009-12-15

**Authors:** Jack W Kent, Charles P Peterson, Thomas D Dyer, Laura Almasy, John Blangero

**Affiliations:** 1 Department of Genetics, Southwest Foundation for Biomedical Research, 7620 NW Loop 410, San Antonio, TX 78227, USA

## Abstract

Many phenotypes may be influenced by the prenatal environment of the mother and/or maternal care, and these maternal effects may have a heritable component. We have implemented in the computer program SOLAR a variance components-based method for detecting indirect effects of maternal genotype on offspring phenotype. Of six phenotypes measured in three generations of the Framingham Heart Study, height showed the strongest evidence (*P *= 0.02) of maternal effect. We conducted a genome-wide association analysis for height, testing both the direct effect of the focal individual's genotype and the indirect effect of the maternal genotype. Offspring height showed suggestive evidence of association with maternal genotype for two single-nucleotide polymorphisms in the trafficking protein particle complex 9 gene *TRAPPC9 *(*NIBP*), which plays a role in neuronal NF-κB signalling. This work establishes a methodological framework for identifying genetic variants that may influence the contribution of the maternal environment to offspring phenotypes.

## Background

Many phenotypes may be influenced by the prenatal environment of the mother and/or maternal care, and these maternal effects may have a heritable component. Much research has focused on the impact of measurable properties of the mother (e.g., adiposity, diabetes, alcohol, or tobacco use) on subsequent phenotypes in their children (e.g., birthweight [1], insulin resistance [2], cognitive function [3]). A more general question is: does the mother's measured **genotype **influence offspring phenotypes, whether or not the intermediate maternal phenotypes are known or measurable? This 'agnostic' (with respect to maternal phenotype) approach has the potential both to identify novel genetic variants of maternal effect and, via 'reverse epidemiology,' to identify novel maternal phenotypes for such effects.

For the purposes of this study, we accept the strict definition of a genetic maternal effect as the indirect effect of maternal genotype on offspring phenotype [4], as distinct from asymmetric transmission of parental alleles (e.g., mitochondrial inheritance [5]) or asymmetric expression of alleles in the offspring depending on parent of origin (e.g., imprinting [6]). Here we develop mixed variance-components models in the computer program SOLAR [7] to estimate maternal random effects on quantitative phenotypes, and use the best such models as null hypotheses for measured-genotype genome-wide association tests of single-nucleotide polymorphism (SNP) genotypes of individuals and their mothers.

## Methods

### Data

Data include adult quantitative phenotypes and Affymetrix SNP genotypes provided in the Genetic Analysis Workshop (GAW) 16 Framingham Heart Study (FHS) data release (Problem 2). All authors of this study are 'approved users' of these data per the NHLBI Data Use Certification of April 2008. Analysis of these data was approved by the Institutional Review Board of the University of Texas Health Science Center, San Antonio.

Outlying phenotype measurements (more than four standard deviations from the mean) were removed from the lipid measures (10 for total cholesterol, 9 for high-density lipoprotein (HDL), 43 for triglyceride (TG)) on the assumption that these represented assay errors. The data were normal-quantile-transformed before analysis using the SOLAR "inormal" option to meet the distributional assumptions of the variance components and regression methods. The normal quantile ("inverse normal") transformation is robust to a range of departures from normality and also removed scale effects by standardizing the data. Transformations of this type are convenient for batch processing of multiple phenotypes (e.g., Peng et al. [8]).

Individuals with incomplete genotype data were given imputed genotype scores for the missing markers using the -- infer option in the computer program Merlin [9,10] Merlin imputes an expected genotype score based on the probability of each possible genotype at a locus given information on marker allele frequency, adjacent markers, and pedigree relationships. We chose not to exclude any SNPs or individuals on the basis of number of incomplete genotypes (unless no genotypes were available at all), given the robustness of imputation from family data [11]. Genotypes were similarly imputed for **all **SNPs for the non-genotyped implicit mothers of genotyped and phenotyped founders. These maternal genotypes entered the association analysis as properties of their offspring (see "Measured genotype analysis," below); the 'virtual' mothers did not enter the analysis otherwise.

### Variance components estimation

We have implemented in SOLAR a general model for incorporating polygenic maternal effects [12-14]. Briefly, in the absence of dominance and epistatic effects, the phenotypic covariance between individuals *i*, *j *may be decomposed into additive genetic and environmental components in the usual way:

where *I*(*i*, *j*) is an identity term (1 if *i *= *j*, or 0 otherwise), *ϕ*(*i*, *j*) is a coefficient of coancestry, *σ*_*z*_(*i*, *j*) is the phenotypic covariance, and *σ*^2^_*e *_and *σ*^2^_*a *_are, respectively, environmental and additive genetic variances. The additive genetic covariance can be further decomposed to include maternal effects:

where 2*ϕ*(*i*, *mo_j*) is the coancestry coefficient for *i *and the mother of *j*, and 2*ϕ*(*mo*_*i*, *mo_j*) is the coancestry of the mothers.  is the additive genetic variance due to maternal effects, and *ρ*_*a*, *am *_is the additive genetic correlation between direct and maternal effects. Decomposition of the environmental component of Eq. 1 is modified from Eq. 14 of Bijma [14]:

with *R *= 1 if *i *= *j *(equivalent to the identity matrix in Eq. 1), *ρ*_*sib *_∈ [0,1] if *i*, *j *are siblings or half-siblings, ρ_mo _∈ [-1,1] if *i*, *j *are mother and offspring, or 0 otherwise. Our modification from Bijma [14] was that twins were not treated differently than other siblings because dizygotic twins could not be distinguished in the de-identified FHS data. Our full mixed model also included the fixed effects of relevant covariates and the random effect of mitochondrial inheritance, *σ*^2^_*mito*_; the mitochondrial variance component is structured by a matrix whose elements are 1 if *i*, *j *belong to the same matriline or 0 otherwise, as described by Czerwinski et al. [15].

### Measured genotype analysis

Measured genotype analysis was conducted for each polymorphic SNP by including its genotype score (the number of copies of the minor allele, range [0,2] with non-integral values for imputed genotypes) as a covariate in the mixed model [16]. Unlike standard association analysis, we included the indirect effect of the mother's genotype in addition to the direct effect of the focal subject's genotype. These effects were tested separately, with an additional test of the mother's genotype conditional on that of the focal subject. The latter test was intended to account for the non-independence of maternal and offspring genotypes: reduction of evidence of maternal association in the conditional test would suggest that the unconditioned maternal effect represented a 'bleed-through' of the direct effect, while an increase in evidence would suggest that the locus affects the trait both directly and indirectly.

## Results

### Screening for evidence of maternal effects

We tested our maternal random effects model on quantitative phenotypes (height, weight, body mass index (BMI), systolic blood pressure (SBP) and diastolic blood pressure (DBP), fasting total and HDL cholesterol and triglycerides) in individuals measured at exams when they were as similar as possible in mean age (Table 1). Sex, age, age^2^, and their interactions were included as covariates in all models, and use of antihypertensive medication was a covariate for SBP and DBP. The use of an indicator variable-type covariate for medication has been questioned, especially with regard to BP [17,18]. In response to a reviewer's concern, we re-ran the BP analyses while correcting for medication by adding 10 mm Hg to BP measures in medicated individuals, as recommended [17]; this did not substantially change our results (data not shown). The impact of alternative corrections for medication may have been greater if we had proceeded to association analysis of the BP traits, because we would then be testing for a difference in means. HDL-C and TG measures were not available for the original cohort and did not give evidence of maternal effect (data not shown); they were not considered further. Results for the remaining phenotypes are given in Table 2.

**Table 1:** FHS cohorts/examination periods used in this study

FHS Cohort/Exam Period	*N*	Age in years [mean (SE)]
Original/Exam 4 (1954-1958)	356	40.9 (0.20)
Offspring/Exam 3 (1983-1987)	2,422	46.3 (0.19)
Generation 3/Exam 1 (2002-2005)	3,997	40.2 (0.14)

**Table 2:** Parameter estimates [mean (SE)] for saturated random-effects model

Trait	σ^2^_a_	σ^2^_am_	ρ_a, am_	σ^2^_e_	ρ_sib_	ρ_mo_	σ^2^_mito_
Height	0.64 (0.02)	0.19 (0.07)	-0.21 (0.16)	0.23 (0.08)	0.03 (0.25)	0.05 (0.29)	0.05 (0.12)
Weight	0.68 (0.03)	0.22 (0.08)	-0.38 (0.16)	0.50 (0.04)	0.00^a^	0.02 (0.12)	0.00 (0.09)
BMI	0.76 (0.04)	0.24 (0.10)	-0.51 (0.14)	0.60 (0.04)	0.00^a^	-0.02 (0.12)	0.00^a^
SBP	0.53 (0.04)	0.04 (0.04)	-1.00^a^	0.74 (0.04)	0.04 (0.03)	0.00 (0.05)	0.00^a^
DBP	0.48 (0.04)	0.24 (0.09)	-0.33 (0.30)	0.80 (0.03)	0.00^a^	0.07 (0.05)	0.00 (0.08)
Cholest.	0.58 (0.05)	0.12 (0.32)	-0.11 (0.75)	0.75 (0.06)	0.05 (0.06)	0.06 (0.07)	0.13 (0.09)

^a^Estimate on boundary; no SE computed.

Table 3 gives the log likelihoods for the minimal polygenic (PG) model (Eq. 1), a PG model with mitochondrial effect, and the saturated model of Table 2. Height showed the strongest evidence of a maternal effect (compared with the PG-mito model, *P *= 0.02 at 4 degrees of freedom; 4 df is probably over-conservative [19]). Interestingly, this trait initially showed a significant mitochondrial effect compared with the PG model (*P *= 0.008, 1 df), evidently capturing some of the maternal effects when these were not explicitly modeled.

**Table 3:** Comparative evidence (log likelihoods), variance-components models

Model	Height	Weight	BMI	SBP	DBP	Cholest.
Polygenic	-112.7	-1850.0	-2788.8	-2460.2	-2724.4	-2615.8
Polygenic + mitochondrial	-109.1	-1850.0	-2788.8	-2460.2	-2724.4	-2613.9
Saturated	-103.3	-1846.9	-2783.5	-2458.4	-2722.7	-2612.8

### Measured genotype analysis

We performed measured genotype (MG) tests of association for own genotype (OMG), maternal measured genotype (MMG), and conditional maternal measured genotype (CMMG), for 476,987 autosomal SNPs from the Affymetrix 500k panel. The saturated maternal effects model was used as the null for all analyses. No SNP gave significant evidence of own- or maternal-genotype association with height when corrected for multiple testing using a Bonferroni test (critical test statistic Λ = 28.374 for genome-wide α = 0.05 and 1 df). We did not attempt to account for any linkage disequilibrium among the SNPs in our sample. The SNPs with strongest evidence for OMG, MMG, and CMMG are listed in Table 4.

**Table 4:** Suggestive associations with height

SNP^a^	Gene	Chrom.	Coordinate (bp)	Λ^b^, OMG	Λ^b^, MMG	Λ^b^, CMMG	MAF^c^	N, typed^d^
Sorted by OMG								
rs2213883	*MPZL1*	1	138,961,675	23.8587	0.8336	0.6778	0.201	6836
rs12129308	*MCOLN2*	1	83,533,561	20.9235	10.6998	3.0661	0.324	6827
rs536609	*MCOLN2*	1	83,540,695	20.8421	11.3493	3.4800	0.325	6786
rs597630	*MCOLN2*	1	83,529,929	20.7087	10.5223	2.9965	0.325	6852
rs600924	*MCOLN2*	1	83,536,001	20.6884	10.5223	3.0128	0.325	6850
Sorted by MMG								
rs11166947	*TRAPPC9*	8	136,376,532	2.3202	24.9813	22.8014	0.322	6271
rs1426022		18	16,635,128	3.7962	22.6984	18.9815	0.468	6675
rs12709669		18	16,653,893	3.3445	22.6099	19.2853	0.467	6838
rs756228	*TRAPPC9*	8	141,138,313	2.5298	21.1932	18.6637	0.31	6841
rs7096364		10	107,344,433	1.8847	21.1904	19.3774	0.112	6849
Sorted by CMMG								
rs11166947	*TRAPPC9*	8	136,376,532	2.3202	24.9813	22.8014	0.322	6271
rs7607015		2	81,548,367	0.3632	21.1517	22.1813	0.077	6670
rs17198973		4	178,164,796	0.3836	16.228	20.2233	0.401	6848
rs11048399	*RASSF8*	12	25,992,998	0.9614	20.2601	19.5277	0.042	6787
rs1195768		10	107,348,297	1.6268	20.201	18.6879	0.118	6553

^a^Likelihood ratio test statistic (Λ = 2 times the difference in log likelihood of test model and its null)

^b^All MG tests have 1 df

^c^MAF = minor allele frequency.

^d^N, typed = number of genotyped individuals (before imputation).

## Discussion

Several recent studies have undertaken genome-wide association analysis of human height [20-22]. These studies have typically examined very large numbers of individuals (~10,000-25,000, with multi-stage designs), larger than the 6,775 individuals in the FHS cohort available for this study. These studies agree in finding numerous loci associated with height, as may be expected for a trait long assumed to be polygenic. Under these circumstances, it is not surprising that we did not replicate specific SNPs or locations identified in these larger studies. It should be noted, however, that we did find suggestive evidence of association (OMG) with a broad genomic region identified by Gudbjartsson et al. [21]: 1q24-25 (Table 4). Our candidate genes in this region is *MPZL1 *(OMIM #604376), a protein tyrosine phosphatase involved in cell proliferation and differentiation. Our next four highest 'hits' were in the mucolipin2 gene *MCOLN2 *on 1p22.

Because the published genome-wide association study on height used unrelated individuals, none reported maternal effects. Interestingly, among our strongest maternal associations are repeated hits in two regions: the *TRAPPC9 *gene on chromosome 8 and an intergenic region on chromosome 18. The trafficking protein particle complex 9 gene *TRAPPC9 *(*NIBP*) plays a role in neuronal NF-κB signalling [23] but has not, to our knowledge, been associated with stature in any published study. The existence of repeated (albeit suggestive) associations in this gene makes it a candidate for further investigation of the effects of maternal genotype on height.

## Conclusion

We have implemented combined random-effects, measured-genotype fixed effects approach for discovery of genetic variants contributing to the indirect effect of maternal genotype on offspring phenotype. We have identified two regions on chromosomes 2 and 8 - with suggestive association at two SNPs in each region - that may contribute to maternal effects on human height. The tools developed here should be of use for a variety of phenotypes and diseases for which an effect of maternal environment is known or suspected, including height, hypertension, birthweight, and the metabolic syndrome.

## List of abbreviations used

BMI: Body mass index; CMMG: Conditional maternal measured genotype; DBP: Diastolic blood pressure; FHS: Framingham Heart Study; GAW16: Genetic Analysis Workshop 16; HDL: High-density lipoprotein; MG: Measured genotype; MMG: Maternal measured genotype; OMG: Own measured genotype; PG: Polygenic SBP: Systolic blood pressure; SNP: Single-nucleotide polymorphism; TG: Triglyceride.

## Competing interests

The authors declare that they have no competing interests.

## Authors' contributions

JB, LA, TDD, and JWK participated in the design of the study. TDD prepared the phenotype and genotype data for analysis. JWK and CPP implemented the maternal-effects analysis in SOLAR. JWK performed the analyses and drafted the manuscript.

## References

[B1] Kazumi T Kawaguchi A Yoshino G Associations of middle-aged mother's but not father's body mass index with 18-year-old son's waist circumferences, birth weight, and serum hepatic enzyme levels Metabolism 2005 54 466 470 10.1016/j.metabol.2004.10.014 15798952

[B2] Gonzalez-Ortiz M Martinez-Abundis E Maternal effect of type 2 diabetes mellitus on insulin sensitivity and metabolic profile in healthy young Mexicans Diabetes Nutr Metab 1999 12 32 36 10517304

[B3] Julvez J Ribas-Fitó N Torrent M Forns M Garcia-Esteban R Sunyer J Maternal smoking habits and cognitive development of children at age 4 years in a population-based birth cohort Int J Epidemiol 2007 36 825 832 10.1093/ije/dym107 17550944

[B4] Hager R Cheverud JM Wolf JB Maternal effects as the cause of parent-of-origin effects that mimic genomic imprinting Genetics 2008 178 1755 1762 18245362 10.1534/genetics.107.080697 PMC2278069

[B5] Yang Q Kim SK Sun F Cui J Larson MG Vasan RS Levy D Schwartz F Maternal influence on blood pressure suggests involvement of mitochondrial DNA in the pathogenesis of hypertension: the Framingham Heart Study J Hypertens 2007 25 2067 2073 10.1097/HJH.0b013e328285a36e 17885549

[B6] Xiong DH Wang JT Wang W Guo YF Xiao P Shen H Jiang H Chen Y Deng H Drees B Recker RR Deng HW Genetic determinants of osteoporosis: lessons learned from a large genome-wide linkage study Hum Biol 2007 79 593 608 18494371 10.1353/hub.2008.0018

[B7] Almasy L Blangero J Multipoint quantitative-trait linkage analysis in general pedigrees Am J Hum Genet 1998 62 1198 1211 9545414 10.1086/301844 PMC1377101

[B8] Peng B Yu RK DeHoff KL Amos CI Normalizing a large number of traits using empirical normal quantile transformation BMC Proc 2007 1 Suppl 1 S156 18466501 10.1186/1753-6561-1-s1-s156 PMC2367615

[B9] Abecasis G Cherny SS Cookson WO Cardon LR Merlin - rapid analysis of dense genetic maps using sparse gene flow trees Nat Genet 2002 30 97 101 10.1038/ng786 11731797

[B10] Burdick JT Chen W-M Abecasis GR Cheung VG *In silico *method for inferring genotypes in pedigrees Nat Genet 2006 38 1002 1004 10.1038/ng1863 16921375 PMC3005330

[B11] Chen W-M Abecasis GR Family-based association tests for genomewide association scans Am J Hum Genet 2007 81 913 926 17924335 10.1086/521580 PMC2265659

[B12] Willham RL The covariance between relatives for characters composed of components contributed by related individuals Biometrics 1963 19 18 27 10.2307/2527570

[B13] Lynch M Walsh B Maternal effects Genetics and Analysis of Quantitative Traits 1998 Sunderland, MA; Sinauer Associates, Inc

[B14] Bijma P Estimating maternal genetic effects in livestock J Anim Sci 2006 84 800 806 16543556 10.2527/2006.844800x

[B15] Czerwinski SA Williams JT Demerath EW Towne B Siervogel RM Blangero J Does accounting for mitochondrial genetic variation improve the fit of genetic models? Genet Epidemiol 2001 21 Suppl 1 S779 S782 11793777 10.1002/gepi.2001.21.s1.s779

[B16] Blangero J Göring HHH Kent JW Jr Williams JT Peterson CP Almasy L Dyer TD Quantitative trait nucleotide analysis using Bayesian model selection Hum Biol 2005 77 541 559 10.1353/hub.2006.0003 16596940

[B17] Cui JS Hopper JL Harrap SB Antihypertensive treatments obscure familial contributions to blood pressure variation Hypertension 2003 41 207 210 10.1161/01.HYP.0000044938.94050.E3 12574083

[B18] Tobin MD Sheehan NA Scurrah KJ Burton PR Adjusting for treatment effects in studies of quantitative traits: antihypertensive therapy and systolic blood pressure Statist Med 2005 24 2911 2935 10.1002/sim.2165 16152135

[B19] Amos C de Andrade M Zhu D Comparison of multivariate tests for genetic linkage Hum Hered 2001 51 133 144 10.1159/000053334 11173964

[B20] Lettre G Jackson AU Gieger C Schumacher FR Berndt SI Sanna S Eyheramendy S Voight BF Butler JL Guiducci C Illig T Hackett R Heid IM Jacobs KB Lyssenko V Uda M Diabetes Genetics Initiative; FUSION; KORA; Prostate, Lung Colorectal and Ovarian Cancer Screening Trial; Nurses' Health Study; SardiNIA Boehnke M Chanock SJ Groop LC Hu FB Isomaa B Kraft P Peltonen L Salomaa V Schlessinger D Hunter DJ Hayes RB Abecasis GR Wichmann HE Mohlke KL Hirschhorn JN Identification of ten loci associated with height highlights new biological pathways in human growth Nat Genet 2008 40 584 591 18391950 10.1038/ng.125 PMC2687076

[B21] Gudbjartsson DF Walters GB Thorleifsson G Stefansson H Halldorsson BV Zusmanovich P Sulem P Thorlacius S Gylfason A Steinberg S Helgadottir A Ingason A Steinthorsdottir V Olafsdottir EJ Olafsdottir GH Jonsson T Borch-Johnsen K Hansen T Andersen G Jorgensen T Pedersen O Aben KK Witjes JA Swinkels DW den Heijer M Franke B Verbeek AL Becker DM Yanek LR Becker LC Tryggvadottir L Rafnar T Gulcher J Kiemeney LA Kong A Thorsteinsdottir U Stefansson K Many sequence variants affecting diversity of adult human height Nat Genet 2008 40 609 615 10.1038/ng.122 18391951

[B22] Weedon MN Lango H Lindgren CM Wallace C Evans DM Mangino M Freathy RM Perry JR Stevens S Hall AS Samani NJ Shields B Prokopenko I Farrall M Dominiczak A Diabetes Genetics Initiative; Wellcome Trust Case Control Consortium Johnson T Bergmann S Beckmann JS Vollenweider P Waterworth DM Mooser V Palmer CN Morris AD Ouwehand WH Cambridge GEM Consortium Zhao JH Li S Loos RJ Barroso I Deloukas P Sandhu MS Wheeler E Soranzo N Inouye M Wareham NJ Caulfield M Munroe PB Hattersley AT McCarthy MI Frayling TM Genome-wide association analysis identifies 20 loci that influence adult height Nat Genet 2008 40 575 583 18391952 10.1038/ng.121 PMC2681221

[B23] Hu W-H Pendergast JS Mo X-M Brambilla R Bracchi-Ricard V Li F Walters WM Blits B He L Schaal S Bethea JR NIBP, a novel NIK and IKK-beta-binding protein that enhances NF-kappa-B activation J Biol Chem 2005 280 29233 29241 10.1074/jbc.M501670200 15951441 PMC3707486

